# A mixed grape and blueberry extract is safe for dogs to consume

**DOI:** 10.1186/s12917-016-0786-5

**Published:** 2016-08-03

**Authors:** Anne-Sophie Martineau, Véronique Leray, Anne Lepoudere, Géraldine Blanchard, Julien Bensalem, David Gaudout, Khadija Ouguerram, Patrick Nguyen, On behalf of Neurophenols Consortium

**Affiliations:** 1 LUNAM University, Oniris, Nantes-Atlantic College of Veterinary Medicine and Food Sciences and Engineering, Nutrition and Endocrinology Unit, C.S. 40706, 44307, Nantes Cedex 03, France; 2 SPF-DIANA Pet Food Business, ZA du Gohélis, 56250, Elven, France; 3 Animal Nutrition Expertise, 33 avenue de l’Île-de-France, 92160, Antony, France; 4 Activ’Inside, Espace Legendre, 33 rue Max Linder, 33500, Libourne, France; 5 UMR1280 Physiologie des Adaptations Nutritionnelles, INRA-Université de Nantes, CHU-Hôtel Dieu, Place Alexis Ricordeau, 44096, Nantes Cedex 1, France

**Keywords:** Dog, Neurophenols, Grape, Blueberry, Kidney, Cystatin C, Clusterin, NGAL, Flavonoids

## Abstract

**Background:**

Grape and blueberry extracts are known to protect against age-related cognitive decline. However, beneficial effects achieved by mixing grape and blueberry extracts have yet to be evaluated in dogs, or their bioavailability assessed. Of concern to us were cases of acute renal failure in dogs, after their ingestion of grapes or raisins. The European Pet Food Industry Federation (2013) considers only the grape or raisin itself to be potentially dangerous; grape-seed extracts *per-se*, are not considered to be a threat. Our aim was therefore to evaluate the renal and hepatic safety, and measure plasma derivatives of a polyphenol-rich extract from grape and blueberry (PEGB; from the Neurophenols Consortium) in dogs. Polyphenol expression was analyzed by UHPLC-MS/MS over 8 hours, for dogs given PEGB at 4 mg/kg. Safety was evaluated using four groups of 6 dogs. These groups received capsules containing no PEGB (control), or PEGB at 4, 20, or 40 mg/kg BW/d, for 24 weeks. Blood and urine samples were taken the week prior to study commencement, then at the end of the 24-wk study period. Routine markers of renal and liver damage, including creatinine (Creat), blood urea nitrogen, albumin, minerals, alkaline phosphatase (ALP), and alanine transaminase (ALT) were measured. Biomarkers for early renal damage were also evaluated in plasma (cystatin C (CysC), and neutrophil gelatinase-associated lipocalin (NGAL)), and urine (CysC, clusterin (Clu), and NGAL). Ratios of urinary biomarkers to Creat were calculated, and compared with acceptable maximal values obtained for healthy dogs, as reported in the literature.

**Results:**

While several PEGB-specific polyphenols and metabolites were detected in dog plasma, at the end of the PEGB consumption period, our biomarker analyses presented no evidence of either renal or liver damage (Creat, BUN, ionogram, albumin and ALT, ALP). Similarly, no indication of early renal damage could be detected. Plasma CysC, urinary CysC/Creat, Clu/Creat, and NGAL/Creat ratios were all beneath reported benchmarked maximums, with no evidence of PEGB toxicity.

**Conclusions:**

Long-term consumption of a pet specific blend of a polyphenol-rich extract from grape and blueberry (PEGB; from the Neurophenols Consortium), was not associated with renal or hepatic injury, and can therefore be considered safe.

## Background

This work comprises part of a project dedicated to the study of age-related cognitive decline in humans and dogs (the Neurophenols Consortium). We aim to complete a novel study into the efficacy of mixed extracts of grape and blueberry in counteracting age-related deterioration of function. In aged dogs, polyphenol ingestion (including grape pomace), and vitamin use, are both thought to ameliorate the effects of aging on learning ability [1]; similarly beneficial effects have been reported for humans [2]. A popular hypothesis is that protection against oxidative stress explains these effects. In aged mice, the consumption of a mixed grape and blueberry extract, has also been shown to improve spatial navigation; one of the skills that declines with age. In this scenario, increased expression for hippocampal nerve growth factor mRNA [3] may play a causal role [4].

Studies reporting grape extract consumption in dogs (using grape seed and skin extracts, or grape seed proanthocyanidins), or grape pomace in aged dogs have, to date, not reported any side effects [1, 5, 6]. However, acute renal failure has been reported in dogs after their consumption of grapes, with kidney histopathology revealing tubular degeneration leading to necrosis, particularly in the proximal tubule [7–9]. In a retrospective study involving a cohort of 43 dogs, all of whom had eaten grapes, raisins, or both, animals presented with clinical signs consistent with kidney deterioration during a window extending from 24 h, until 5 days, after consumption. Vomiting, diarrhea, lethargy, and either olig- or, anuria, were the common clinical signs. A diagnosis of renal damage was supported by biochemical abnormalities showing higher plasma creatinine (Creat), blood urea nitrogen (BUN), an altered ionogram, glycosuria, and proteinuria. Again, histopathology revealed severe diffuse renal tubular degeneration, especially in proximal cells, with glomerular deterioration. Half of the dogs died [9]. In these cases, the precise amount of fruit eaten varied greatly (from 3 g/kg BW of raisin, to 150 g/kg BW of grape), as did the type of fruit (grape, raisin, seedless grape), and the affected breed [7–9]. Hepatic toxicity has also been associated with the consumption of plants such as greater celandine, green tea, valerian, or ayurvedic products. In these cases, higher concentrations of alanine transaminase (ALT), alkaline phosphatase (ALP), aspartate aminotransferase (AST), and bilirubin, were all demonstrated (reviewed in [10, 11]). Abnormal values of ALT and ALP provoked by grape consumption also point to the liver being a target of grape toxicity [9], although the factors responsible for hepatic damage, as well as the acute renal failure, have yet to be identified.

The Neurophenols Consortium is a Europe-North America research collaboration dedicated to the research, and development of natural ingredients and products to prevent age-related cognitive decline in humans and pets. The Consortium brings together scientists in the fields of phytochemistry, neuroscience, psychology and nutrition with companies specialized in the development of active ingredients and food supplements. The specific aims of the program are to characterize and formulate fruit extracts from blueberry and grape, to evaluate their safety and efficacy in pre-clinical and clinical trials.

The aim of this study was to assess the safety of a polyphenol-rich extract from grape and blueberry (PEGB; from the Neurophenols Consortium). We studied the safety of this extract following chronic use in dogs, by monitoring renal and hepatic health, using early biomarkers of renal damage as well as a biochemical approach.

## Methods

### Animals

Twenty-four experimental Beagle dogs (4 groups of 5 males, and a single female, body condition score (BCS) 5/9, mean age 31 ± 3 months, mean body weight (BW) 11.4 ± 0.2 kg), originally from CEDS (Centre d’élevage du Domaine des Souches, Mézilles, France), were used. They were fed with a dry maintenance diet (Medium Adult Royal Canin), according to the National Research Council (NRC 2006) [12] recommendation (130 kcal metabolizable energy per kg metabolic body weight).

### Study design

Four groups of 6 dogs (each comprising 5 males and a female) were given a polyphenol-rich extract from grape and blueberry (PEGB) for 24 weeks. The constituents of this PEGB extract were devised by the Neurophenols Consortium; these were grape (*Vitis vinifera L*.), and blueberry (*Vaccinium angustifolium*) extracts, containing specific polyphenols with low molecular weight monomers, including catechin (6 % dry matter), oligomers, flavonols (for a total of 0.15 % dry matter), anthocyanins, phenolic acids, and resveratrol formulated in a unique ratio of molecules. The intended dosage was 4 mg/kg BW/d. One group also received a control dose of 0 mg/kg BW/d (control), with two other groups receiving higher doses of the extract; 20 mg/kg BW/d, and 40 mg/kg BW/d. Each dose was given in the daily meal as a gelatin capsule (Cooper, Melun Cedex, France) containing the formulation and maltodextrin. All extracts were prepared in accordance with good laboratory practices.

### Plasma and urine samples

Blood and urine samples were collected in the week prior to the study commencing, and then at the end of the 24-wk period. Blood samples were obtained by jugular venipuncture into heparin tubes in 24-hour unfed animals. Each blood draw was immediately centrifuged (2124 g for 10 min at 4 °C), and the plasma fraction aliquoted and frozen at -80 °C. Twenty-four-hour urine samples were collected by voiding, following the consumption of the daily meal and the capsules. The expression of specific polyphenols, derived from the PEGB extract, were measured, on the 8th days of exposure, in plasma samples from dogs that were given PEGB at 4 mg/kg/d. For this purpose, plasma samples were taken for 8 h, with polyphenol analyses performed by UHPLC-MS/MS.

### Chemical analyses

Concentrations of Creat, BUN, minerals (sodium (Na^+^), potassium (K^+^), calcium (Ca^2+^), and phosphate (PO_4_^3-^), albumin, ALT, and ALP, were determined using a VetScan reagent rotor (Comprehensive Diagnostic Profile, VetScan VS2, Abaxis, Ca, USA). Biomarkers of renal damage, including CysC (cystatin C), Clu (clusterin), and NGAL (neutrophil gelatinase-associated lipocalin), were measured by species-specific ELISA (canine cystatin C, Biovendor, Czech Republic; canine clusterin, Biovendor, Czech Republic; dog NGAL, Bioporto, Denmark). Urinary Creat was assayed using an enzymatic colorimetric kit (Creatinine, Randox Laboratories, UK).

### Data analyses

Results are reported as means ± standard error of the mean (SEM). For each early biomarker of renal damage, we compared their maximal values after PEGB consumption with previous maximal values reported for healthy dogs. As replicate datasets were collected, linear mixed-effects model analyses could be undertaken to investigate any interaction between PEGB dose, and time. Moreover, an inter-group analysis was performed using a linear model to compare data for each of the experimental groups (4, 20, and 40 mg/kg/d), with the control group, at the beginning and end of the study. Finally, an intra-group analysis was completed using a linear mixed effects model to compare data from the initiation and end of the study. These analyses were completed using the R software (R Core Team (2013)). The alpha level for determination of significance was 0.05.

## Results

### Specific polyphenols in plasma following PEGB consumption

Polyphenols and their metabolites were detected in plasma samples, and their maximum concentrations (Cmax) determined. These metabolites comprised: hydroxy and dihydroxyphenyl-γ-valerolactone, both derived from flavan-3-ols; the resveratrol derivatives, reseveratrol glucuronide, dihydroresveratrol sulfate, and glucuronide; the flavonol and its metabolite, quercetin and isorhamnetine sulfate; and the anthocyanin metabolite, malvidin. A Cmax for the flavan-3-ol metabolites of 2028 nM was attained after 8 h. Flavonol metabolites reached a Cmax of 5nM, also after 8 h, with malvidin also peaking (7nM) at this timepoint. Peak concentrations for resveratrol metabolites were reached much earlier, after 30 min (Cmax 161 nM).

### Plasma and urine biomarkers

#### Markers of liver damage

Plasma hepatic biomarker concentrations are shown in Table 1. All ALT and ALP concentrations were within the reference range.

**Table 1:** Plasma biomarkers of kidney damage in dogs, at the initiation (Week -1) and the end (Week 24) of a 24-wk period of consumption of PEGB at 4, 20 or 40 mg/kg/d. (Data are means ± SEM, *n* = 6/group; minima and maxima values are mentioned in parenthesis)

Biomarkers (reference values)	Units	Control		PEGB					
				4 mg/kg/d		20 mg/kg/d		40 mg/kg/d	
		Week -1	Week 24	Week -1	Week 24	Week -1	Week 24	Week -1	Week 24
Creatinine (0.3–1.4)	mg/dL	0.8 ± 0.1 (0.4–1.0)	0.8 ± 0.1 (0.5–1.0)	0.9 ± 0.1 (0.7–1.1)	0.7 ± 0.1 (0.6–0.9)	0.8 ± 0.1 (0.5–1.1)	0.8 ± 0.1 (0.5–1.3)	0.9 ± 0.1 (0.7–1.1)	0.8 ± 0.1 (0.5–0.9)
Blood urea nitrogen (7–25)	mg/dL	14 ± 1 (11–15)	13 ± 1 (10–15)	12 ± 1 (8–15)	11 ± 1 (7–14)	10 ± 1 (8–10)	9 ± 1 (7–13)	12 ± 1 (10–17)	12 ± 1 (9–15)
Sodium (138–160)	mmol/L	145 ± 2 (138–150)	142 ± 2 (137–147)	146 ± 2 (138–152)	142 ± 2 (136–148)	147 ± 1 (143–151)	142 ± 1 (138–146)	145 ± 1 (143–147)	146 ± 2 (140–151)
Potassium (3.7–5.8)	mmol/L	4.7 ± 0.1 (4.4–5.1)	4.7 ± 0.3 (3.7–5.4)	4.4 ± 0.1 (3.8–4.7)	4.6 ± 0.2 (3.6–5.0)	4.5 ± 0.1 (4.2–4.9)	4.5 ± 0.2 (3.9–5.1)	4.6 ± 0.1 (4.4–4.9)	4.8 ± 0.3 (4.0–5.8)
Calcium (8.6–11.8)	mg/dL	10.1 ± 0.2 (9.4–10.5)	10.1 ± 0.1 (9.8–10.7)	10.7 ± 0.2 (9.8–11)	10.4 ± 0.1 (10.0–10.8)	10.5 ± 0.3 (9.6–11.2)	10.4 ± 0.2 (9.8–10.8)	10.5 ± 0.1 (10.1–10.9)	10.5 ± 0.1 (10.1–10.8)
Phosphate (2.9–6.6)	mg/dL	4.3 ± 0.2 (3.6–5.0)	4.2 ± 0.3 (3.3–4.9)	4.5 ± 0.3 (3.9–5.8)	4.4 ± 0.3 (3.3–5.2)	4.2 ± 0.2 (3.4–5.1)	4.4 ± 0.3 (3.6–5.1)	4.4 ± 0.2 (3.4–5.1)	4.2 ± 0.1 (3.8–4.6)
Albumin (2.5–4.4)	g/dL	3.1 ± 0.2 (2.3–3.4)	3.2 ± 0.2 (2.6–3.6)	3.5 ± 0.1 (3.2–3.6)	3.3 ± 0.1 (2.7–3.6)	3.4 ± 0.2 (2.9–3.4)	3.3 ± 0.2 (2.3–3.9)	3.4 ± 0.1 (3.0–3.6)	3.4 ± 0.1 (3.3–3.7)

#### Markers of renal damage

Plasma creatinine, urea, sodium, potassium, calcium, phosphate, and albumin concentrations are shown in Table 2. All values were found to be in the reference range, i.e. the 95 % prediction interval (for a normal population).

**Table 2:** Plasma biomarkers of liver damage in dogs, at the initiation (Week -1) and the end (Week 24) of a 24-wk period of consumption of PEGB at 4, 20 or 40 mg/kg/d. (Data are means ± SEM, *n* = 6/group; minima and maxima values are mentioned in parenthesis)

Biomarkers (reference values)	Units	Control		PEGB					
				4 mg/kg/d		20 mg/kg/j		40 mg/kg/j	
		Week -1	Week 24	Week -1	Week 24	Week -1	Week 24	Week -1	Week 24
Alanine transaminase (10–118)	U/L	38 ± 5 (27–59)	36 ± 5 (26–57)	51 ± 7 (26–60)	44 ± 9 (20–76)	45 ± 2 (39–52)	46 ± 39 (38–61)	41 ± 5 (28–60)	45 ± 4 (28–60)
Alkaline phosphate (20–150)	U/L	59 ± 9 (33–88)	63 ± 10 (32–86)	63 ± 6 (49–86)	65 ± 12 (51–126)	55 ± 9 (37–88)	57 ± 14 (33–109)	48 ± 7 (29–78)	49 ± 9 (20–80)

The concentration of early renal biomarkers, and their ratios, are presented in Table 3. For each biomarker, interaction analyses failed to identify any difference between the experimental and control groups, either at the beginning, or at the end of the study. No inter-group or intra-group variations could be noted between experimental groups, compared to controls, or between the initiation and the end of the study.

**Table 3:** Concentrations, and ratios, of early biomarkers of renal damage in dogs, at the initiation (Week -1) and the end (Week 24) of a 24-wk period of consumption of PEGB at 4, 20 or 40 mg/kg/d. (Data are means ± SEM, *n* = 6/group; minima and maxima values are mentioned in parenthesis)

				PEGB					
Biomarkers	Units	Control		4 mg/kg/d		20 mg/kg/d		40 mg/kg/d	
		Week -1	Week 24	Week -1	Week 24	Week -1	Week 24	Week -1	Week 24
Plasma CysC	μg/mL	1.2 ± 0.1 (1.0–1.6)	1.1 ± 0.1 (0.7–1.7)	1.3 ± 0.2 (0.6–1.7)	1.2 ± 0.2 (0.5–1.8)	1.2 ± 0.1 (0.9–1.5)	1.2 ± 0.1 (0.9–1.4)	1.5 ± 0.2 (1.1–2.2)	1.3 ± 0.1 (0.9–1.6)
Urinary CysC/Creat ratio	μg/g	19 ± 17 (0–105)	14 ± 10 (0–62)	5 ± 4 (0–20)	4 ± 1 (0–9)	27 ± 14 (3–96)	21 ± 12 (3–79)	12 ± 4 (2–26)	4 ± 1 (0–9)
Urinary Clu/Creat ratio	ng/g	79 ± 20 (7–134)	135 ± 66 (6–443)	44 ± 13 (3–99)	94 ± 69 (10–437)	63 ± 28 (6–170)	56 ± 25 (12–175)	84 ± 23 (18–163)	47 ± 20 (9–132)
Urinary NGAL/Creat ratio	ng/g	7.6 ± 3.0 (0.6–21.1)	7.2 ± 2.5 (1.2–16.5)	7.9 ± 4.5 (0.6–28.5)	4.9 ± 1.4 (0.9–9.5)	4.2 ± 1.8 (1.0–12.8)	3.5 ± 0.3 (2.5–4.0)	5.4 ± 1.0 (2.3–9.4)	4.6 ± 1.4 (1.8–10.0)

**Fig. 1:**
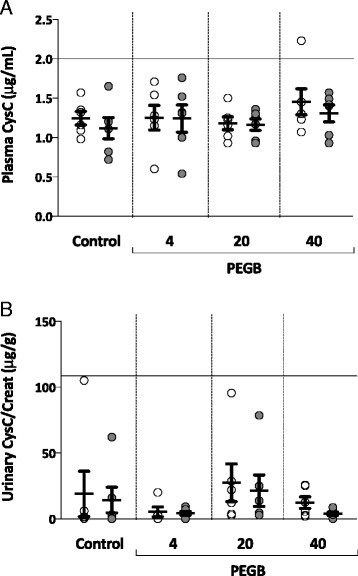
**a** Plasma Cystatin C concentration (μg/mL) in dogs at the initiation () and the end () of a 24-wk period of consumption of PEGB at 4, 20 or 40 mg/kg/d (*n* = 6 dogs per group). The line indicates the reported maximal value in normal dogs. **b** Urinary Cystatin C/Creatinine ratio (μg/g) in dogs, at the initiation () and the end () of a 24-wk period of consumption of PEGB at 4, 20 or 40 mg/kg/d (*n* = 6 dogs per group). The line indicates the reported maximal value in normal dogs

**Fig. 2:**
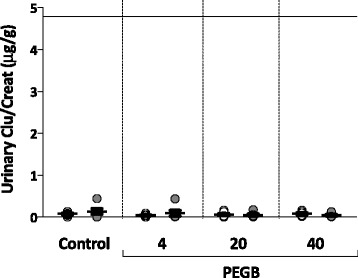
Urinary Clusterin/Creatinine ratio (ng/g) in dogs, at the initiation () and the end () of a 24-wk period of consumption of PEGB at 4, 20 or 40 mg/kg/d (*n* = 6 dogs per group). The line indicates the reported maximal value in normal dogs

**Fig. 3:**
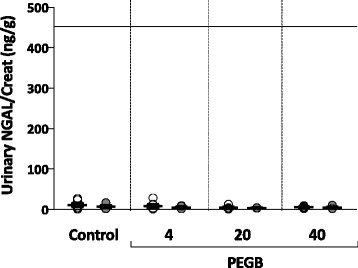
Urinary NGAL/Creatinine ratio in dogs (ng/g) before at the initiation () and the end () of a 24-wk period of consumption of PEGB at 4, 20 or 40 mg/kg/d (*n* = 6 dogs per group). The line indicates the reported maximal value in normal dogs

In the experimental groups (PEGB given at 4, 20 or 40 mg/kg/d), the mean plasma CysC concentrations were found to be similar to control group. Mean concentrations ranged from 1.2 to 1.5 μg/mL (Fig. 1a). Intra-group analyses also showed no differences across the experimental groups. Collectively, their mean urinary CysC/Creat ratio varied from 4 to 27 μg/g. These ratios were not significantly different to those determined for the control group, for whom no intra-group difference was noted (Fig. 1b). Mean urinary Clu/Creat ratios varied between 44 and 94 ng/g in groups given PEGB at 4 to 40 mg/kg/d, again with no significant changes compared to control group nor intra-group differences between the initiation and the end of the study (Fig. 2). The mean urinary NGAL/Creat ratios were similar to control group in the experimental groups, varying between 4 and 8 ng/g. Intra-group analyses also failed to determine any significant differences between the initiation and the end of the study (Fig. 3).

## Discussion

Our aim was to assess the safety of a polyphenol-rich extract from grape and blueberry (PEGB; from the Neurophenols Consortium) for dogs, by monitoring early biomarkers of renal damage over a 24-week period. This work considerably extends the previous study periods reported, where platelet effects, and gene expression profiles, were interrogated after 7 days, or 3 months of supplement use [5, 6].

After PEGB consumption, biomarker values exceeded the reported maximal limits in no dog, with no differences observed at the end of the 24-week period, compared to beginning, for plasma CysC, and urinary CysC/Creat, Clu/Creat, or NGAL/Creat ratios. When considering these data, we conclude that the dogs neither presented with renal, nor hepatic injury, at the end of the study.

While bioavailability of the Neurophenols Consortium PEGB had never been evaluated in dogs, our evaluation of the safety of this supplement necessitated measurement of PEGB derivatives in plasma. The main polyphenols in the extract were flavan-3-ols, resveratrol, anthocyanins (malvidin, petunidin, peonidin, petunidin, cyanidin), and flavonol (quercetin). Some polyphenols and polyphenol metabolites were found in plasma. Malvidin, which is present in blueberry but not in grape, has been the only anthocyan detected, but it is known that anthocyanins are less absorbed than other flavonoids. The finding of resveratrol derivatives (which are grape specific) is in accordance with a study that also showed appearance of resveratrol conjugates (sulfate & glucuronide) in the plasma of dogs after resveratrol administration [13]. The valerolactones detected resulted from the metabolization of flavan-3-ols by gut microflora. Quercetin and isorhamnetin sulfate, which are present in both fruits, were also found. Other compounds may have been absorbed, but either they have not been identified, or their concentration was under the detection threshold, or they were rapidly metabolized and excreted. Very few data on polyphenols pharmacokinetics in dogs are available. Regarding resveratrol, Cmax could not be compared since in previous report [13] it was given to dogs at much higher doses than the intended dose in the present study (200–1200 mg/kg/d, compared to 4 mg/kg/d). When anthocyanins were given to pigs at 1 to 4 % of the diet (w/w), several metabolites were measured in liver, eye and brain while there were not detected in plasma [14], and again the doses were far higher than in the present study. Catechin and epicatechin glucuronides from a grape extract given to mice were measured in plasma [15], which was not the case in our study, but the dose used was still much higher (grape-derived polyphenols: 80 mg/kg/d). When green tea catechins (13 mg/kg/d, [16], 170 mg/kg/d [17]) and epigallocatechin gallate (EGCG; 250 mg/kg/d [18]) were given to dogs, respective metabolites were found in plasma, which was not the case after PEGB consumption where only valerolactones were detected. The difference could be explained either by the catechin sources or higher doses or both. Another possible explanation is that dogs were given the PEGB at the same time of their daily meal, and the plasma measurements were done after a relatively short period of exposure. Indeed in dogs given EGCG at 300 mg/kg/d, plasma area under the curve (AUC) for EGCG was higher in unfed than fed dogs [19]. When EGCG was given at 500 mg/kg/d, authors reported, although the difference did not reach the significance level, that the AUC for EGCG was 1.6 time higher after 28 days of dosing than after 14 days [19]. The data of the present study demonstrated that the polyphenols of the PEGB extract were, at least in part, bioavailable, and this is the first report on the appearance of valerolactones as well as quercetin, isorhamnetin sulfate and malvidin in the plasma of dogs after consumption of a mixture of polyphenols.

The origin of the grape toxicity described in the literature for dogs is still obscure, but numerous hypotheses have emerged. Among them, it was reported that exogenous compounds on grapes, such as mycotoxin, pesticides, or herbicide residues, could be responsible for the kidney toxicity, with histopathology indicating that the proximal cells are the primary target [8]. These findings provoked further hypotheses, such as the toxic accumulation of a foreign chemical (a xenobiotic), with a particular affinity for tubular specific transporters. Additionally, the expression of a perinuclear golden brown pigment [8], could imply its cytotoxic accumulation, with failed cellular clearance. Hypercalcemia and renal mineralization induced by the high sugar content of grapes are also current hypotheses.

The resveratrol concentration in grapes could also be responsible for renal damage. A previous study described that the no-observed-adverse-effect level of resveratrol consumption was 600 mg/kg BW/d in dogs. Consumption of twice this dose (1200 mg/kg BW/d) induced a loss of appetite, and weight [20]. Given that grapes contain 1.5 to 7.8 μg of total resveratrol per gram of fresh weight [21], it is highly unlikely that resveratrol is responsible for the acute kidney injury observed in clinical cases in dogs.

Plasma creatinine and urea are the most frequently measured parameters used to evaluate renal damage. High creatinine concentrations are seen when at least 75 % of renal function has already been lost [22]. In previous studies describing acute renal failure after grape consumption, symptoms appeared rapidly [9]. Therefore, we reasoned that to monitor kidney health, earlier biomarkers of renal damage would be required. In 2010, the Nephrotoxicity Working Group established a consortium between the European Medicines Agency, and the Food and Drug Administration. They listed seven biomarkers needed to detect the early development of renal injury [23]. Among these, we chose to assess CysC and Clu, because of their ease of use in dogs. In addition, NGAL was measured, as a promising early biomarker of drug-induced kidney injury. Collectively, these early biomarkers of renal damage are ideal for monitoring renal health, before irreversible damage, as they survey different renal functions, and compartments of the kidney.

Ordinarily, cystatin C, which is a low molecular weight protein produced at a constant rate by all cells, is completely reabsorbed and catabolized in proximal tubular epithelial cells [24]. Following renal injury, CysC concentration increases in the plasma, as the glomerular filtration rate declines [25]; an increased concentration in urine reflects tubular impairment [26]. Plasma CysC has previously been measured in healthy dogs (urea and creatinine concentrations within reference intervals), with the highest reported values of 2 μg/ml [27]. For all dogs that had received PEGB, at any dose, plasma CysC concentrations were beneath this upper limit. To the best of our knowledge, the referenced study [27] is the only one in which plasma CysC concentrations have been measured in healthy dogs by canine ELISA. We therefore conducted the same tests, in our study. In other studies, CysC was measured in serum and/or with a different ELISA kit or technique (i.e. Particle-Enhanced Turbidimetric Immunoassay), which may explain the slightly different reference ranges reported [28–30]. In our study, the maximum urinary CysC/Creat ratio that we measured in dogs following PEGB consumption (regardless of dose) was 79 μg/g, whereas reported urinary CysC/Creat ratios have been as higher as 0.11 ± 0.02 mg/g [31]. Therefore, we conclude that our CysC results revealed no glomerular or tubular impairments.

Clusterin is a high molecular weight glycoprotein expressed in epithelial cells (reviewed in [32]); in cases of acute renal failure, clusterin is found at high concentrations in the urine, indicating glomerular damage [33]. The highest urinary Clu/Creat ratio previously reported in healthy dogs was 4.87 μg/g [33]. In this study, the urinary Clu/Creat ratio measured in dogs after PEGB consumption (4 to 40 mg/kg/d) was far lower, ranging from 10 to 437 ng/g. Therefore, clusterin analyses also revealed no evidence of glomerular damage after PEGB consumption.

NGAL is a protein that has raised some interest since its mRNA and protein were detected in urine after induction of acute kidney injury in rodents [34]. NGAL mRNA has been found in the ascending limb of Henle, and in collecting duct cells after ischemia-reperfusion [35]. NGAL is ordinarily reabsorbed by the proximal tubule [35, 36]. However, in case of renal injury, reabsorption may decrease, which results in higher urinary concentrations. Tubular damage and reduced filtration may also cause the accumulation of plasma NGAL [37]. The reported ranges of urinary NGAL/Creat ratio have varied greatly in healthy dogs from 10 to 460 ng/g, or from 40 to 3660 ng/g [38, 39]. These variations could reflect reporting from client-owned dogs of various breeds, age, and gender, fed with various diets. In our study, the urinary NGAL/Creat ratios after PEGB consumption (at any dose), ranged from 0.9 to 10 ng/g, leading us to conclude that there was no evidence of tubular damage. Recently, it was found that plasma NGAL was not an absolute criterion with which to discriminate between a healthy dog, versus a dog with either chronic, or acute kidney disease [38] contrary to urinary NGAL [39] and this shows how we must be cautious when interpreting these values. Moreover, increasing plasma NGAL would reflect tubular and filtration dysfunction, data already provided by other early biomarkers of renal damage (Plasma CysC, and urinary CysC/Creat, NGAL/Creat, and Clu/Creat ratios). Therefore, we suggest that plasma NGAL measurements represent redundant data and can be omitted.

Intermediate measurements were also taken during the 24-week study period for all biomarkers; these did not reveal any significant differences.

The PEGB doses ranged from 4 to 40 mg/kg/d, the intentional dose for dogs facing cognitive decline being 4 mg/kg/d [as recommended by the Neurophenols Consortium]. In studies where dogs were fed supplements with grape seed/skin extract at 20 mg/kg/d [5], or grape seed proanthocyanidins at 5 mg/kg/d [6], symptoms related to acute renal failure were not reported. In the group given the PEGB at 4 mg/kg/d, the dose of grape extract was beneath these previously reported doses. In addition, dogs consuming five or even ten times the intentional PEGB dose, showed no alteration of kidney or hepatic damage at 24 weeks. These data corroborated the 2013 European Pet Food Industry Federation (FEDIAF) advice that dogs could safely consume grape extract.

We have considered why our extract, consumed long-term, as described in this study, appears to be entirely safe for consumption by dogs, in stark contrast to reports of acute renal failure in pets following their consumption of whole grapes or raisins. We can envisage some possibilities. The extract developed by our consortium is actually a complex mix of different extracts. How these extracts are derived (i.e. extracted from the grape), may have reduced, denatured, or eliminated, potentially toxic compounds. These factors may underlie the lack of any discernable toxicity when dogs consume the Neurophenols Consortium extract, even at high doses.

## Conclusions

Following consumption of the PEGB at all doses, conventional biomarkers of renal and liver damage were within the reference range throughout the study, with values of early biomarkers of renal damage CysC, Clu, NGAL unremarkable. To our knowledge, this is the first study demonstrating that chronic consumption of the PEGB extract can be achieved with neither renal, nor hepatic damage, at least based on plasma and urine analyses. Of note, renal health was monitored using a panel of parameters encompassing both early biomarkers of renal damage, as well as conventional biochemistry; this complementary approach is recommended in future studies. To conclude, dogs can safely consume a polyphenol-rich extract from grape and blueberry (PEGB; from the Neurophenols Consortium).

## Abbreviations

ALP, Alkaline phosphatase; ALT, Alanine transaminase; AST, Aspartate aminotransferase; AUC, Area under the curve; BCS, Body condition score; BUN, Blood urea nitrogen; BW, Body weight; Clu, Clusterin; Cmax, maximum concentrations; Creat, Creatinine; CysC, Cystatin C; EGCG, Epigallocatechin gallate; NGAL, Neutrophil gelatinase-associated lipocalin; PEGB, Polyphenol-rich extract from grape and blueberry; UHPLC-MS/MS, Ultra high-performance liquid chromatography coupled to tandem mass spectrometry
